# Associations between adult ADHD core symptoms, cognitive flexibility, and emotional eating: a case-control study

**DOI:** 10.3389/fpsyt.2026.1761915

**Published:** 2026-04-02

**Authors:** Selin Karakaya, Bedriye Öncü

**Affiliations:** Department of Psychiatry, Ankara University Faculty of Medicine, Ankara, Türkiye

**Keywords:** attention deficit/hyperactivity disorder (ADHD), cognitive flexibility, eating disorders, emotional eating, executive function, inattention

## Abstract

**Introduction:**

Attention-deficit/hyperactivity disorder (ADHD) in adults often co-occurs with eating disorders (EDs), potentially through shared difficulties in emotional regulation, and executive functions. This study explored the associations between cognitive flexibility as a component of executive functions, core adult ADHD symptom dimensions and emotional eating-related eating behaviorsin adults with ADHD and healthy controls, within the framework of executive functions.

**Methods:**

This case-control study included 76 adults with ADHD and 69 healthy controls. Participants completed the Self-Report Wender-Reimherr Adult Attention Deficit Disorder Scale (SR-WRAADDS), Emotional Eating Questionnaire (EEQ), Hospital Anxiety and Depression Scale, Cognitive Control and Flexibility Questionnaire (CCFQ), and Berg’s Card Sorting Test. Group differences were tested with t-tests, correlations with Spearman’s ρ, and hierarchical regression (Approval No: I11-798-23).

**Results:**

The ADHD group had significantly higher EEQ scores (t = 5.39, p =0.001). The ADHD group also showed lower CCFQ total score (t (125) = –5.52, p <0.001). EEQ scores were positively correlated with SR-WRAADDS Attention Deficit (ρ =0.331, p =0.003), and CCFQ Cognitive Control over Emotion (ρ = −0.256, p =0.02). Regression analysis identified attention deficit as the only significant predictor of the EEQ total scorein the ADHD group.

**Discussion:**

Our findings suggest that impairments in executive functioning—including cognitive flexibility, attentional regulation, and emotion-related control mechanisms—may play a more central role in the relationship between ADHD and emotional eating-related eating behaviors. Longitudinal studies are warrented to further elucidate these mechanisms.

## 1 Introduction

In adulthood, attention deficit/hyperactivity disorder (ADHD) is characterized not only by inattention, hyperactivity, and impulsivity (1). In individuals with ADHD, executive functions (EFs)which are higher-order neurocognitive processes critical for guiding and regulating attention, affect, and action, are also known to be impaired (2). Although the composition of EFs is debatable, it is generally agreed that core EFs include working memory, response inhibition, planning, and cognitive flexibility (CF) (3–5), which is defined as the capacity to recognize changing circumstances and adapt behavior by evaluating alternative options (6, 7).

Although EFs have traditionally been viewed as purely cognitive skills elicited under relatively abstract, decontextualized, and non-affective conditions, the role of motivation and emotion in EF has become increasingly important in recent years (8). This traditional EF construct is referred to as “cold” EF, which involves logical reasoning, critical analysis, and conscious control of behavior without an affective component (9), In contrast, “hot” EFs involve goal-directed cognitive processes that arise in contexts characterized by emotional and motivational influences, particularly when there is a conflict between immediate gratification and long-term rewards (10, 11). “Hot” EF has been proposed to encompass emotional cognitive abilities such as delay of gratification and emotional decision-making (12).

Executive function impairments may substantially contribute to the high prevalence of psychiatric comorbidities observed in individuals with ADHD, among which eating disorders (EDs) constitute a particularly relevant and clinically significant comorbidity (13). Regarding the often comorbidity of EDs in adults with ADHD, EF impairments have been shown to substantially influence maladaptive eating behaviors (14). Core ED symptoms encompass heightened concerns about eating and weight, body image disturbances, and maladaptive compensatory behaviors, including self-induced vomiting (15). This rigid behavioral pattern may reflect the impaired CF, as a component of EFs, commonly observed in individuals with EDs. Although some inconsistencies exist in the literature, deficits in CF have been reported in individuals diagnosed with anorexia nervosa (AN) and other specified eating disorders, and these impairments appear to be independent of illness duration or severity (16–19). In addition, although CF impairments in bulimia nervosa (BN) have been investigated less extensively than in AN and the existing findings remain somewhat inconsistent, several studies suggest that individuals with BN may experience difficulties particularly in domains of CF (20, 21). Similarly, impaired CF may be associated with disordered eating behaviors characterized by more severe loss of control, as well as with binge eating disorder (21–23).

Another shared clinical feature of adult ADHD and EDs is emotion dysregulation. Emotional dysregulation refers to excessive emotional reactivity (e.g., rapid mood changes and the emergence of high-intensity emotions disproportionate to the trigger or context) as well as deficits in emotion regulation (e.g., an inability to control these emotional responses through appropriate emotion regulation strategies) (24). Although knowledge about the neurocognitive and behavioral mechanisms underlying the association between ADHD and emotional dysregulation remains limited (25), it has been suggested that emotional dysregulation in ADHD may be closely related to deficits in EFs, given that emotion regulation involves strategies such as inhibition, reappraisal, and suppression (26). Such dysregulation ——naturally leads to the concept of emotional eating which refers to the tendency to overeat in response to negative emotional states such as anxiety or irritability (27). In the context of EDs, emotional eating may reflect broader difficulties in emotion regulation (28).

The common traits of individuals with ADHD and ED, such as impaired EF, emotional dysregulation and impulsivity, suggest that shared mechanisms such as CF may play a role in their clinical presentation. Although CF has been investigated in relation to both ADHD and EDs separately, the interplay between these two conditions has rarely been examined from the perspective of CF. Given their frequent co-occurrence and their potential to contribute to significant mental and physical health problems, understanding the underlying mechanisms of this association is critically important.

This study aimed to investigate the relationship between emotional eating-related eating behaviors and CF in adults diagnosed with ADHD, within the framework of EFs, and to examine the relationships between core adult ADHD characteristics and emotional eating. A healthy control group was also included to enable for both between-group comparisons and within-group analyses. In this context, the study sought to explore potential factors that may influence emotional eating-related eating behaviors.

## 2 Materials and methods

The study included 76 adults diagnosed with ADHD and 69 healthy controls recruited from the Department of Psychiatry, Ankara University Faculty of Medicine. The required sample size was calculated using G*Power 3.1.9.2 software (29), and which indicated that at least 64 participants per group were necessary to achieve 95% confidence, 80% power, and a 5% margin of error.

The ADHD group comprised who presented to the outpatient clinics of the Department of Psychiatry at Ankara University Faculty of Medicine and were diagnosed with ADHD according to the DSM-5 criteria. This group included individuals who had an established diagnosis of ADHD based on a clinical interview and were already under follow-up at our clinic. Inclusion criteria for the ADHD group were age between 18 and 65 years and having a DSM-5 diagnosis of ADHD. The exclusion criteria were intellectual disability (IQ <80), a specific learning disorder, schizophrenia or schizoaffective disorder, bipolar disorder in an active phase, and a current major depressive episode. Intellectual disability and specific learning disorder were excluded based on clinical assessment, including psychiatric examination, developmental and educational history, and review of available medical records, rather than formal psychometric testing. Initially, 78 individuals with ADHD were evaluated, of whom two were excluded for not meeting the inclusion criteria, resulting in a final sample of 76 participants.

The healthy control group was formed through convenience sampling. Inclusion criteria were age between 18 and 65 years and literacy, with no diagnosis of ADHD. Exclusion criteria included active psychopathology or use of psychiatric medication Although 77 individuals without a diagnosis of ADHD were initially recruited, eight were excluded due to psychiatric comorbidities. Thus, the final control group comprised 69 adults who met the inclusion criteria. The authors confirm that all procedures contributing to this work comply with the ethical standards of the relevant national and institutional committees on human experimentation and with the Helsinki Declaration of 1975, as revised in 2013. All procedures involving human subjects were approved by the Ethics Committee of Ankara University Faculty of Medicine (Approval No: I11-798-23). Participants using stimulant medication were instructed to withhold methylphenidate for at least 8 hours prior to neurocognitive testing. Stimulant treatment in our sample consisted exclusively of methylphenidate formulations, with no amphetamine-based stimulants. Importantly, participants who had used long-acting methylphenidate formulations on the day of testing were not included in the neurocognitive assessment and were instead assessed on a separate day, whereas the 8-hour washout criterion was applied only to those using immediate-release methylphenidate. Participants were included in the study only after an interval of at least 8 hours without alcohol, or nicotine-containing substances prior to testing to minimize acute effects on cognitive performance.

All participants were recruited on a voluntary basis and provided written informed consent after receiving detailed information about the study. Following completion of a sociodemographic data form, participants underwent the computerized version of the Wisconsin Card Sorting Test (WCST), known as the 64-card Berg’s Card Sorting Test (BCST). They then completed self-report questionnaires: the Wender-Reimherr Adult Attention Deficit Disorder Scale (SR-WRAADDS), the Emotional Eating Questionnaire (EEQ), the Hospital Anxiety and Depression Scale (HADS), and the Cognitive Control and Flexibility Questionnaire (CCFQ). Each participant took part in a single session conducted in a clinical setting, which lasted approximately 30 minutes.

### 2.1 Tools

#### 2.1.1 Sociodemographic data form

This form was developed by the researchers to collect information on participants’ sociodemographic characteristics; physical conditions; current medications; body mass index (BMI); use of tobacco, alcohol, and other substances; and behaviors potentially indicative of eating disorders.

#### 2.1.2 Self – Report Wender–Reimher Adult Attention Deficit Disorder Scale

he SR-WRAADDS is a 61-item, 5-point Likert-type self-report scale that assesses ADHD symptoms in adults based on the Wender–Utah criteria, with particular emphasis on evaluating emotional dysregulation (30). The Turkish validity and reliability study of the SR-WRAADDS was conducted by Karakaya et al. The scale comprises four subscales: emotional dysregulation, attention deficit, impulsivity/hyperactivity, and temperamental traits. The self-report version of the scale was found to be valid and reliable. The internal consistency coefficients of the SR-WRAADDS ranged from 0.72 to 0.90 (31).

#### 2.1.3 Emotional Eating Questionnaire

The Emotional Eating Questionnaire was developed by Garaulet et al., and its Turkish adaptation and validation were conducted by Arslantaş et al. The scale consists of 10 items rated on a 4-point Likert scale and includes three subscales: inability to control eating, type of food consumed, and feelings of guilt (32). In the original version, scores between 11 and 20 indicate emotional eating, whereas scores of 21 or above indicate highly emotional eating. Internal consistency coefficients ranged from 0.59 to 0.87 (33).

#### 2.1.4 Hospital Anxiety and Depression Scale

The Hospital Anxiety and Depression Scale is a brief self-report measure designed to quickly and reliably assess levels of anxiety and depression (34). A Turkish adaptation and validity–reliability study were conducted, which demonstrated that the scale is suitable for Turkish culture (35).

#### 2.1.5 Cognitive Control and Flexibility Questionnaire

The Cognitive Control and Flexibility Questionnaire is a scale developed to assess individuals’ levels of cognitive control and flexibility in response to stressful situations (36). It was adapted into Turkish. The 18-item, seven-point Likert scale with a two-factor structure (Cognitive Control over Emotion and Appraisal and Coping Flexibility) demonstrated good reliability, with Cronbach’s alpha coefficients ranging from 0.85 to 0.91 (37).

#### 2.1.6 Berg’s Card Sorting Test

Developed by Heaton et al., the Wisconsin Card Sorting Test is administered via two separate decks consisting of four stimulus cards and 64 response cards (38). It primarily assesses abstraction, planning, mental flexibility, working memory, and monitoring abilities. The test was adapted into Turkish and was found to be valid and reliable (39). In the present study, the computerized version of the WCST, known as the Berg Card Sorting Test, was used. The BCST provides 12 different outcome scores. Among these, the number and percentage of perseverative responses and perseverative errors are grouped under the domain of perseveration and are considered key indicators of CF (40).

### 2.2 Statistical analyses

All the statistical analyses were performed using SPSS version 24.0 for Windows (SPSS Inc., Chicago, IL, USA). Descriptive statistics are presented as frequencies, percentages, means, standard deviations, medians, minimums, and maximum values. The chi-square test was used to compare categorical variables. Due to low expected frequencies in several BMI categories, between-group differences were examined using the Fisher–Freeman–Halton exact test. The normality of continuous variables was assessed via visual methods and skewness–kurtosis values (values between –1.5 and +1.5 were considered acceptable) (41). Depending on normality, continuous variables were compared via Student’s t test or the Mann–Whitney U test. Given that 40 independent t-tests were conducted, a Bonferroni correction was applied. The adjusted significance threshold was set at p < 0.001.

Group differences in EEQ total scores were additionally examined using ANCOVA, with diagnostic group as the fixed factor and sex, HADS anxiety, HADS depression, and BMI as covariates (two-tailed tests; partial η² reported).

Correlations between continuous variables were analyzed using Spearman’s correlation test for non-normally distributed data. Additionally, partial correlation analyses were performed to examine the associations between EEQ total scores and selected SR-WRAADDS and CCFQ subscales while controlling for potential confounding variables. BMI, HADS anxiety and depression subscales, were included as control variables in these analyses. All partial correlation analyses were conducted separately for the ADHD and healthy control groups using two-tailed tests.

To examine the factors associated with emotional eating, a hierarchical multiple linear regression analysis was conducted with the EEQ total score as the dependent variable. Prior to running the hierarchical model, the key regression assumptions—including normality, homoscedasticity, multicollinearity, outliers, and autocorrelation—were evaluated. Multicollinearity was assessed using tolerance and variance inflation factor (VIF) values, all of which were found to be within acceptable limits (tolerance>0.10; VIF <10).

In Model 1, age and sex were entered as control variables. In Model 2, all clinical predictors were added to determine whether they explained additional variance in emotional eating beyond age and sex. The overall model significance at each step was assessed using ANOVA, and effect sizes were reported using R² and adjusted R² values. The Durbin–Watson statistic was 2.003, indicating no evidence of autocorrelation in the model. All analyses were conducted at the 95% confidence level, and statistical significance was set at p < 0.05.

## 3 Results

### 3.1 Sociodemographic and clinical characteristics of the participants

A total of 145 participants were included in the study, comprising 76 individuals with ADHD and 69 healthy controls. The mean age of the ADHD group was 23.28 years, with the majority being female (59.2%). In the control group, the mean age was 23.08 years, and the majority were female (56.5%). No statistically significant differences were found between the groups in terms of age, sex, marital status, or educational level. The income level did not follow a normal distribution and was analyzed via the Mann–Whitney U test. No significant difference was observed between the two groups (U = 1586.50, Z =-0.50, p =0.61). Additionally, two participants in the ADHD group had a diagnosis of bipolar affective disorder; however, they were not in an active episode during the study period. The presence of physical illness (18.4% in the ADHD group vs. 10.1% in the control group) did not differ significantly between the groups. One of the eating disorder symptoms—self-induced vomiting following episodes of uncontrolled eating—was also significantly more prevalent in the ADHD group compared with the healthy control group. The sociodemographic and clinical characteristics of the participants are summarized in **Table 1**.

**Table 1:** Sociodemographic, clinical characteristics of the ADHD and control groups.

Variables	ADHD Group (n = 76)	Control Group (n = 69)	Statistical Analysis	p value
Age				0.77¹
Mean (SD)	23.28 (4.64)	23.08 (3.56)	t = 0.292	
Range	18–42	19–44	df = 143	
Gender, n (%)				0.74²
Female	45 (59.2%)	39 (56.5%)	χ² = 0.10	
Male	31 (40.8%)	30 (43.5%)	df = 1	
Marital Status, n (%)				0.47²
Single	72 (94.7%)	67 (97.1%)	χ² = 0.51	
Married	4 (5.3%)	2 (2.9%)	df = 1	
Education Level, n (%)				0.44²
Primary School	1 (1.3%)	—		
Secondary School	—	1 (1.4%)		
High School	37 (48.7%)	29 (42.0%)	χ² = 2.65	
University	38 (50.0%)	39 (56.5%)	df = 3	
Smoking, n (%)				0.002²
Yes	27 (35.5%)	9 (13.0%)	χ² = 9.79	
No	49 (64.5%)	60 (87.0%)	df = 1	
Alcohol Use, n (%)				0.01²
Yes	42 (55.3%)	25 (33.2%)	χ² = 5.64	
No	33 (43.4%)	44 (63.8%)	df = 1	
Substance Use, n (%)				0.01²
At least once	6 (7.9%)	0 (0.0%)	χ² = 5.68	
Never	70 (92.1%)	69 (100.0%)	df = 1	
Physical Illness, n (%)				0.15²
Yes	14 (18.4%)	7 (10.1%)	χ² = 2.00	
No	62 (81.6%)	62 (89.9%)	df = 1	
Self-induced Vomiting After Binge Eating, n(%)				0.005²
Yes	13 (17.1%)	2 (2.9%)	χ² = 7.87	
No	63 (82.9%)	67 (97.1%)	df = 1	
Coresidence Status, n (%)			—	—
Dormitory/Institution	2 (2.6%)	9 (13.2%)		
Alone	16 (21.1%)	15 (21.7%)		
With Family	54 (71.1%)	40 (58.0%)		
With Roommates	4 (5.3%)	4 (5.8%)		
Occupation, n (%)			—	—
Unemployed	5 (6.6%)	2 (2.9%)		
Student	55 (72.4%)	59 (85.5%)		
Homemaker	1 (1.3%)	1 (1.4%)		
Worker	1 (1.3%)	2 (2.9%)		
Officer	8 (10.5%)	2 (2.9%)		
Other	6 (7.9%)	3 (4.3%)		

¹Independent samples t test, ²Pearson’s chi-square test, SD, standard deviation; significance was set at *p* < 0.05.

In the ADHD group, 77.6% of participants were receiving psychiatric medication. The most commonly used medication type was stimulants (40.8%), followed by combination pharmacotherapy (28.9%), antidepressants (5.3%).

**Figure 1:**
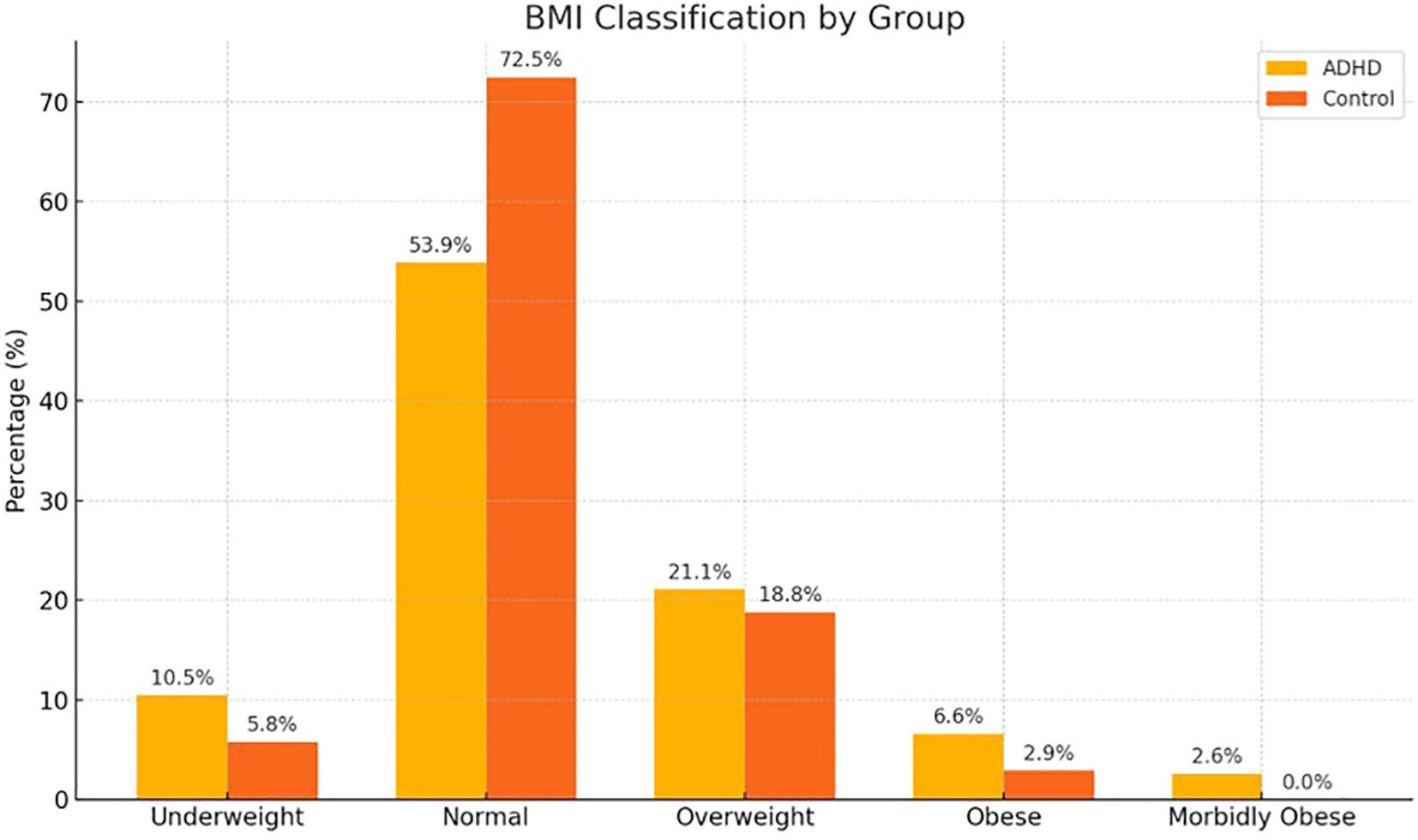
Illustrates the distribution of BMI categories in the ADHD and control groups using a bar chart.

The distribution of BMI categories did not significantly differ between the ADHD and control groups (Fisher–Freeman–Halton exact test, two-sided p = .290). Although the ADHD group included a higher proportion of individuals classified as underweight, obese, or morbidly obese, these differences did not reach statistical significance. The detailed distribution is presented in **Figure 1**.

### 3.2 Sex differences in sociodemographic and clinical variables

Sex differences were examined using independent samples t-tests for all measured parameters within groups. Among the healthy controls, a significant difference was found only in BMI between women and men, with men having higher BMI scores (female: *M* = 21.09 ± 2.14; male: *M* = 24.73± 3.99; *t* (41) = −4.5, *p* < 0.001). No significant sex differences were observed (all p> 0.05).

### 3.3 Group differences in emotional eating and cognitive flexibility

Independent samples t-tests revealed significant group differences across all measures. The ADHD group had a higher EEQ total score (15.44 ± 7.62) compared with the control group (9.21 ± 6.26), indicating greater emotional eating tendencies, t (141) = 5.39, p <0.001, with a large effect size (Cohen’s d =0.89). In contrast, the ADHD group scored lower on the CCFQ Cognitive Control over Emotion score subscale (58.34 ± 4.82) relative to controls (60.76 ± 4.02), t (143) = –3.26, p = 0.001, corresponding to a small-to-medium effect size (Cohen’s d = –0.42). Similarly, the ADHD group showed significantly lower scores on the CCFQ Appraisal and Cognitive Flexibility subscale (41.48 ± 11.96) compared with the control group (50.46 ± 6.57), t (118) = –5.66, p =0.001, with a large effect size (Cohen’s d =–0.88). These results remained statistically significant after Bonferroni correction for multiple comparisons.

An ANCOVA was conducted with EEQ total score as the dependent variable, diagnostic group (ADHD vs. healthy control) and sex as fixed factors, and HADS anxiety, HADS depression, and BMI as covariates. The overall model was significant (F(5,135) = 13.62, p < 0.001), explaining 33.5% of the variance in EEQ scores (R² = 0.335). Significant effects were observed for HADS anxiety (F(1,135) = 14.71, p < 0.001, partial η² = 0.098), BMI (F(1,135) = 23.80, p < 0.001, partial η² = 0.150), sex (F(1,135) = 5.27, p = 0.023, partial η² = 0.038), and group (F(1,135) = 5.02, p = 0.027, partial η² = 0.036), whereas HADS depression was not significant (F(1,135) = 0.01, p = 0.923).According to the BCST scores, group comparisons of perseverative responses and errors indicated that the ADHD group had higher scores on both measures. The number of perseverative errors was significantly greater in the ADHD group (M = 8.64 ± 5.87) than in the control group (M = 7.99 ± 10.43), with a Mann–Whitney U value of 2105.00, *p* = 0.03, and a Z score of −2.06. Although the number of perseverative responses was also higher in the ADHD group (M = 18.38 ± 8.41) than in the control group (M = 16.58 ± 6.35), this difference did not reach statistical significance (*p* = 0.06).

### 3.4 Correlations in the ADHD group

Normality analyses were conducted using the Shapiro–Wilk test. The EEQ total score, did not follow a normal distribution (p =0.033). In the ADHD group, Spearman correlation analysis revealed that the EEQ total score was significantly correlated with the SR-WRAADDS Attention Deficit subscale (ρ = 0.331, p = 0.003). In contrast, the EEQ total score showed no significant correlations with the SR-WRAADDS Impulsivity/Hyperactivity (ρ =0.140, p =0.22), Emotion Dysregulation (ρ =0.210, p =0.06), or Temperamental Traits subscales (ρ = 0.094, p =0.41).

Upon evaluation of CF, within the ADHD group, Spearman correlation analysis revealed that a significant negative correlation was observed between the EEQ total score and the CCFQ Cognitive Control over Emotion score (ρ =−0.256, *p* = 0.02). Although the correlations between the total EEQ scores and the CCFQ Appraisal and Coping Flexibility scores were also negative, they did not reach statistical significance (ρ =−0.22, *p* = 0.05). Additionally, no significant correlations were observed between performance scores on the BCST perseverative responses and errors, and the total EEQ score within the ADHD group (*p* = 0.47 and *p* = 0.59).

Within the ADHD group, correlation analyses revealed that HADS anxiety scores were significantly associated with the EEQ total score (ρ =0.291, p =0.01). However, no significant correlation was found between HADS depression scores and the EEQ total score (ρ =0.103, p= 0.37).

Additionally, in the ADHD group, partial correlation analyses controlling for HADS anxiety score, HADS depressive score, and BMI showed that EEQ scores remained significantly associated with both the SR-WRAADDS attention deficit subscale (ρ = 0.33, p = 0.006) and the CCFQ Cognitive Control over Emotion subscale (ρ = −0.26, p = 0.029).

### 3.5 Within-group comparisons in the ADHD group

Within the ADHD group, individuals scoring 11 or above on the EEQ (classified as emotional eaters) were compared to those scoring below 11 with respect to SR-WRAADDS, HADS, and CCFQ subscale scores as well as BMI, using independent samples t-tests. When applying the Bonferroni-adjusted significance threshold (α_adj =0.0013) for multiple comparisons, none of the group differences between emotional eaters and non-emotional eaters within the ADHD group remained statistically significant after correction. The findings are summarized in **Table 2**.

**Table 2:** Comparison of emotional eaters and non-emotional eaters within the ADHD group.

Measure	EEQ ≥ 11 (n = 54) Mean (SD)	EEQ < 11 (n = 22) Mean (SD)	Statistical test	Effect size (Cohen’s d)
SR-WRAADDS – Emotion Dysregulation	39.50 (7.99)	35.09 (10.36)	t(74) = 1.99 p = 0.05	0.47
SR-WRAADDS – Attention Deficit	34.62 (5.83)	29.54 (8.50)	t(74) = 2.99 p = 0.004	0.69
SR-WRAADDS – Impulsivity/Hyperactivity	20.38 (5.54)	18.59 (6.02)	t(74) = 1.25 p = 0.21	0.30
SR-WRAADDS – Temperamental Traits	12.62 (4.55)	11.36 (4.49)	t(74) = 1.10 p = 0.27	0.27
HADS – Anxiety	11.92 (4.52)	8.86 (4.74)	t(74) = 2.64 p = 0.01	0.66
HADS – Depression	7.42 (3.79)	6.45 (3.86)	t(74) = 1.00 p = 0.31	0.25
CCFQ – Cognitive Control over Emotion	57.59 (4.52)	60.18 (5.16)	t(74) = -2.17 p = 0.03	-0.53
CCFQ – Appraisal and Cognitive Flexibility	39.33 (12.33)	46.77 (9.26)	t(74) = -2.54 p = 0.01	-0.68
BMI	24.39 (5.47)	21.87 (3.66)	t(70) = 1.90 p = 0.06	0.54

EEQ, Emotional Eating Questionnaire; SR-WRAADDS, Self-Report Wender-Reimhers Adult Attention Deficit Disorder Scale; HADS, Hospital Anxiety and Depression Scale; CCFQ, Cognitive Control and Flexibility Questionnaire; BMI, Body Mass Index; SD, Standard Deviation *p*, significance level. Significance was set at *p* < 0.001. (These results were not significant after Bonferroni correction).

Although SR-WRAADDS Attention Deficit and Emotion Dysregulation subscales scores were higher in emotional eaters (t =2.99, p =0.004, and t =1.99, p =0.05 respectively), these differences did not reach the corrected significance threshold. Similarly, elevations in HADS Anxiety scores (t = 2.64, p =0.01), HADS Depression scores (t =1.00, p =0.25) and differences on the CCFQ Cognitive Control over Emotion (t =−2.17, p =-0.03) and CCFQ Appraisal and Coping Flexibility subscales (t =−2.54, p =0.012) were no longer significant after correction. No significant differences were observed in BMI.

Additionally, no significant differences were found in BCST perseverative errors or responses between emotional eaters and non-emotional eaters within the ADHD group (p =0.82 and p =0.63 respectively).

As the final step of the within-group analyses conducted in the ADHD sample, emotional eating and CF scores were compared between individuals who reported compensatory vomiting following episodes of uncontrolled eating and those who did not, using independent samples t-tests. The participants with compensatory vomiting (n =13) demonstrated higher total scores on the EEQ (M = 21.15 ± 6.82) than did those without such behavior (n =63; M = 14.26 ± 7.28), *t* (74) =-3.13, *p* = 0.002, *Cohen’s d* = -0.954. Regarding CF, individuals without vomiting behavior scored higher on the CCFQ Appraisal and Coping Flexibility subscale (*t* (74) = 2.20, *p* = 0.03, *d* = 0.67). Although individuals who reported compensatory vomiting following episodes of uncontrolled eating showed higher scores on several measures, these results did not reach statistical significance.

No significant differences were also found between groups in terms of the CCFQ Cognitive Control over Emotion subscale (*p* = 0.13) and SR-WRAADDS subscales scores (p >0.05) and BCST scores (*p >*0.05), which were compared using the Mann–Whitney U test (p >0.05).

### 3.6 Hierarchical regression predicting emotional eating in the ADHD group

A hierarchical multiple linear regression analysis was conducted with the EEQ total score as the dependent variable. In the first step of the hierarchical regression analysis (Model 1), age and sex were entered as control variables. The model was not statistically significant (R = 0.187, R² = 0.035, Adjusted R² = 0.009, F (2, 73) = 1.327, p =0.272), indicating that these demographic factors explained only 3.5% of the variance in ADHD group. The Durbin–Watson statistic was 1.983, suggesting no autocorrelation.

In Model 2, all clinical variables were added after controlling for age and sex. Multicollinearity diagnostics (VIF and tolerance) indicated no problematic collinearity among predictors, with VIF values ranging from 1.44 to 4.24 and tolerance values between 0.23 and 0.69. Model 2 explained 20% of the variance in EEQ scores (R = 0.447, R² =0.200, Adjusted R² =0.104). The increase in explained variance was statistically significant (ΔR² = .165, F-change (8, 67) = 2.091, p =0.049). Among all predictors, only the SR-WRAADDS Attention Deficit subscale remained a significant predictor of total EEQ score. Detailed regression coefficients are presented in **Table 3**.

**Table 3:** Hierarchical regression analysis (Model 2): regression coefficients for predictors of total EEQ score.

Predictor	B	SE	β	t	p
Constant	22.44	14.70	—	1.52	0.13
SR-WRAADDS – Emotional Dysregulation	-0.61	0.19	-0.71	-0.31	0.75
SR-WRAADDS – Attention Deficit	0.49	0.20	0.45	2.45	0.01
SR-WRAADDS – Hyperactivity/Impulsivite	-0.09	0.22	-0.07	-0.42	0.67
SR-WRAADDS – Temperamental traits	-0.32	0.27	-0.19	-1.21	0.23
HADS – Anxiety	0.10	0.24	0.06	0.43	0.66
HADS – Depression	0.05	0.26	0.02	0.20	0.83
CCFQ –Cognitive Control over Emotion	-0.22	0.23	-0.14	-0.97	0.33
CCFQ – Appraisal and Coping Flexibility	-0.83	0.09	-0.13	-0.89	0.37

SR, WRAADDS, Self-Report Wender-Reimherr Adult Attention Deficit Disorder Scale; HADS, Hospital Anxiety and Depression Scale; CCFQ, Cognitive Control and Flexibility Questionnaire; EEQ, Emotional Eating Questionnaire. *B*, unstandardized coefficient; *SE*, standard error; *β*, standardized coefficient; *t*, t statistic; *p*, significance level. Significance was set at *p* < 0.05.

### 3.7 Correlations in the control group

In the healthy control group, Spearman correlation analysis indicated. The EEQ total score was significantly associated with certain adult ADHD symptom dimensions. Specifically, the EEQ total score was positively correlated with the SR-WRAADDS Emotion Dysregulation subscale (ρ =0.416, p <0.001), the SR-WRAADDS Impulsivity/Hyperactivity subscale (ρ =0.303, p = 0.011) and the SR-WRAADDS Temperamental traits (ρ =0.214, p =0.014), whereas no significant correlation was observed with the SR-WRAADDS Attention Deficit subscale (ρ =0.15, p =0.21). Additionally, EEQ total scores were negatively correlated with the CCFQ Cognitive Control over Emotion subscale (ρ =–0.334, p =0.005) and with the CCFQ Appraisal and Coping Flexibility subscale (ρ =–0.296, p =0.014).

In the control group, similar to the ADHD group, the EEQ total score was not correlated with the HADS Depression subscale score (ρ =0.151, p =0.214) but was significantly positively correlated with the HADS Anxiety subscale score (ρ =0.354, p =0.003).

In the healthy control group, partial correlation analyses controlling for HADS anxiety, HADS depressive symptoms, and BMI showed that EEQ total scores were not significantly associated with the SR-WRAADDS Emotion Dysregulation (r = 0.14, p = 0.255), Impulsivity/Hyperactivity (r = 0.20, p = 0.103), or Temperamental Traits (r = 0.07, p = 0.591) subscales, nor with the CCFQ Cognitive Control over Emotion (r = −0.21, p = 0.086) or Appraisal and Coping Flexibility (r = −0.21, p = 0.092) subscales.

### 3.8 Within-group comparisons in the healthy control group

The participants in the control group were categorized as *emotional eaters* or *non-emotional eaters*. Independent samples t tests were subsequently conducted to compare symptom severity, CF, mood-related symptoms, and BMI between these subgroups. Regarding EEQ total score, emotional eaters demonstrated significantly higher scores on measures of SR-WRAADDS Emotion Dysregulation (*t*(67) = 3.49, *p* = 0.00, *d* = 0.85), Impulsivity/Hyperactivity (*t*(32) = 2.68, *p* = 0.01, *d* = 0.70), Temperamental traits (*t*(44) = 2.47, *p* = 0.00, *d* = 0.74). BMI was also significantly greater in this subgroup (*t* (67) =3.02, *p* < 0.001, *d* = 0.74). These results were not significant after Bonferroni correction. In terms of CF, a difference was observed in the CCFQ Appraisal and Coping Flexibility subscale (*t*(67) =-2.38, *p* = 0.02, *d* =−0.57), indicating reduced flexibility among emotional eaters however, this difference did not remain statistically significant after applying the Bonferroni correction. No significant group differences were found for the SR-WRAADDS Attention Deficit (*t*(67) =-1.41, *p* = 0.16, *d* =−0.34), CCFQ Cognitive Control Over Emotion (*t(*67*)* = 1.79, *p* = 0.07, *d* = 0.44), HADS-Depression (*t*(67) = -1.70, *p* = 0.09, *d* =−4.18), or HADS Anxiety (*t*(67) = -2.05, *p* = 0.04, *d* =−0.50) scores.

## 4 Discussion

The primary aim of this study was to examine the relationship between emotional eating*-related eating behaviors*and CF, within the framework of both “hot” and “cold” EFs, as well as adult ADHD symptom dimensions within an ADHD sample. Consistent with expectations, our findings demonstrated that individuals with ADHD showed markedly reduced CF compared with healthy controls, as reflected by lower score on the CCFQ and higher perseverative error rates on the BCST. This finding is consistent with previous literature (42–44).

Firstly, within the ADHD group, emotional eating showed a significant association only with adult inattention symptoms, while no meaningful correlations were observed with other ADHD variables. In our results, EEQ total scores were correlated with both HADS Anxiety and the CCFQ Cognitive Control over Emotion subscale. Following partial correlation analyses controlling for anxiety, depression, and BMI, the associations between EEQ scores and both the attention deficit subscale of SR-WRAADDS and the Cognitive Control over Emotion subscale remained statistically significant in the ADHD group. In contrast, when the same analyses were conducted in the healthy control group, none of the associations that were initially significant retained statistical significance after adjustment. Secondly, when participants were categorized as emotional eaters versus non–emotional eaters based on EEQ cut-off scores, no significant group differences were observed across any of the measured variables after correction. In contrast, among individuals with ADHD, those who reported self-induced vomiting exhibited significantly lower CCFQ flexibility scores. This pattern suggests that CF may play a more critical role in compensatory behaviors such as vomiting, rather than in emotional eating itself. Nevertheless, this finding should be interpreted with caution, as the vomiting subgroup within the ADHD group comprised a relatively small sample size.

Another noteworthy finding of our study was that, in the regression analysis, inattention emerged as the only significant predictor of emotional eating. Interestingly, this pattern was not observed in the healthy controls, in whom other ADHD-related symptom domains showed significant associations in both the correlation analyses and within-group comparisons, whereas attention deficit did not. In addition, although the differences in mean BMI values were not statistically significant, the lower proportion of individuals with a normal BMI in the ADHD group, as illustrated in **Figure 1**, together with their higher EEQ scores, suggests a higher prevalence of disordered eating tendencies such as emotional eating in this population. These results may indicate that attentional processes play a particularly relevant role in shaping eating behaviors among adults with ADHD. There is growing evidence that cognitive processes such as attention to food and the encoding and retrieval of memories related to recently consumed foods play an important role in appetite regulation by influencing meal size and the interval between meals (45, 46). Distraction from food during eating may weaken memory traces related to the amount consumed, which in turn may lead to increased subsequent intake. Indeed, the meta-analysis conducted by Robinson et al. highlights the central role of attention and memory in the regulation of eating behavior (47). The prominence of the inattention dimension in the ADHD group observed in the present study may be grounded in this mechanism. Nevertheless, it is important to note that attentional functioning reflects an individual’s capacity to selectively allocate attention (48, 49) and is closely related to other EF components, especially “hot” EFs (50). Such patterns were supported by evidence of altered performance on emotion-related decision-making tasks in obesity (51, 52).

On the other hand, research on the relationship between emotion regulation and EFs in adult ADHD samples is limited and has yielded inconsistent findings (53). Emotional dysregulation in ADHD has been linked to shared self-regulatory deficits affecting both cognitive and emotional regulation, which are also commonly observed in EDs and may stem from impairments in physiological arousal, inhibitory control, attentional regulation, and action planning processes (54, 55). In this context, the observed association between emotional eating and the Cognitive Control over Emotion subscale in the ADHD group may further reflect the involvement of emotion-related executive control processes in eating behaviors.

Impairments in EFs in ADHD may be related to deficits in attention and other cognitive processes, rather than to a direct impairment in CF (13). It has been suggested that the primary mechanism underlying impaired CF is inattention, which sequentially leads to difficulties in processing, retaining, and recalling information (7). Impaired CF and a reduced capacity for emotional control may hinder the regulation of intrusive negative thoughts under stress, thereby triggering automatic, rapid, and maladaptive responses (38, 56). This observation is consistent with prior research on EFs in EDs, which has consistently demonstrated impairments in decision-making, response inhibition, and CF (57, 58). Moreover, a recent cohort study conducted in children and adolescents highlighted the potential role of CF in the development of EDs among youth diagnosed with ADHD (59, 60).

When these interrelated regulatory processes are considered collectively, the concept of “hot” EFs becomes particularly salient for understanding the regulation of dysfunctional behaviors and thoughts involving attention, inhibitory control, and CF. Evidence from eating disorder research supports this framework with task-based studies reporting impairments in decision-making, set-shifting, and attentional control under emotionally salient conditions in individuals with EDs. For example, performance deficits have been observed on the Iowa Gambling Task and Emotional Stroop Task in individuals with AN or BN, including those in remission (61, 62). In contrast, some studies using modified Stroop paradigms have not identified significant differences between individuals with BN and healthy controls (63).

Taken together, these findings may help contextualize our results, in which associations were primarily observed with the subjective CCFQ, a measure more closely related to “hot” EFs and emotion-related regulatory processes. In contrast, no significant associations were detected with the BCST, which predominantly assesses “cold” EFs under emotionally neutral conditions. This pattern suggests that the observed associations may reflect the combined influence of emotion regulation difficulties, inattention, and other interrelated executive processes, rather than isolated impairments in CF captured by performance-based tasks. Emotional eating-related eating behaviors may also be linked to the complex and heterogeneous nature of ADHD, common psychiatric comorbidities or shared genetic factors (64, 65). Given the frequent comorbidity of ADHD with disorders that have both psychiatric and physical consequences, understanding the underlying mechanisms is critically important.

Among the strengths of our study are the comparable sociodemographic characteristics between groups—including age, sex, education level, and income—which help minimize potential confounding effects on the results. Partial correlation and ANCOVA analyses were conducted as supportive analyses, allowing the robustness of the observed associations to be examined while controlling for potential confounding variables. In addition, the present study integrated both subjective and objective measures of CF, providing a more comprehensive assessment of EF. Importantly, the inclusion of both “cold” and “hot” EF domains enabled the examination of differential patterns of significance across these systems, as reflected in the study findings.

However, several limitations should be acknowledged. ADHD subtypes, comorbid conditions, and the effects of medication use could not be evaluated in relation to the outcomes. Despite existing recommendations suggesting a 24-hour washout period for methylphenidate (66, 67), the washout duration in the present study was determined pragmatically to enhance participant compliance: 8 hours following short-acting formulations and around 24 hours for long-acting formulations. Accordingly, residual pharmacological effects of methylphenidate cannot be entirely excluded and should be considered when interpreting the results. Similarly, although participants were instructed to maintain short-term abstinence from alcohol and nicotine prior to assessment, residual or cumulative effects related to habitual use may still have influenced cognitive performance. As this was a single-center study, the findings should be interpreted with consideration of potential cultural influences. The predominance of female participants may also have influenced eating-related outcomes, given the higher prevalence of emotional eating among women, and the results should therefore be interpreted with caution. The ADHD group primarily consisted of young adults, which was expected given that most clinical referrals originate from this age range. To minimize potential age-related bias, the control group was matched to a similar age distribution. The very small number of ADHD participants reporting self-induced vomiting limits the interpretability of this behavior. The objective assessment of CF was limited to the BCST, and a significant between-group difference was observed only in perseverative error scores. No significant correlations were found between BCST scores and emotional eating, which is consistent with prior studies indicating the test’s limited specificity (68, 69). This study has a cross-sectional case–control design; therefore, causal inferences cannot be drawn from the results.

Inattention, cognitive flexibility, and other interrelated executive processes may represent an important mechanism in the link between ADHD and emotional eating-related eating behaviors, although the complex and often comorbid nature of ADHD could also contribute to this association. Further longitudinal research is needed to better understand these pathways.

## Data Availability

The raw data supporting the conclusions of this article will be made available by the authors, without undue reservation.
